# Slow naming of pictures facilitates memory for their names

**DOI:** 10.3758/s13423-019-01620-x

**Published:** 2019-06-13

**Authors:** Eirini Zormpa, Antje S. Meyer, Laurel E. Brehm

**Affiliations:** 1 Psychology of Language Department, Max Planck Institute for Psycholinguistics, P.O. Box 310, 6500 AH Nijmegen, the Netherlands; 2 Radboud University Nijmegen, P.O. Box 9102, 6500 HC Nijmegen, the Netherlands

**Keywords:** Word production, Long-term memory, Generation effect, Processing time

## Abstract

Speakers remember their own utterances better than those of their interlocutors, suggesting that language production is beneficial to memory. This may be partly explained by a generation effect: The act of generating a word is known to lead to a memory advantage (Slamecka & Graf, 1978). In earlier work, we showed a generation effect for recognition of images (Zormpa, Brehm, Hoedemaker, & Meyer, 2019). Here, we tested whether the recognition of their names would also benefit from name generation. Testing whether picture naming improves memory for words was our primary aim, as it serves to clarify whether the representations affected by generation are visual or conceptual/lexical. A secondary aim was to assess the influence of processing time on memory. Fifty-one participants named pictures in three conditions: after hearing the picture name (identity condition), backward speech, or an unrelated word. A day later, recognition memory was tested in a yes/no task. Memory in the backward speech and unrelated conditions, which required generation, was superior to memory in the identity condition, which did not require generation. The time taken by participants for naming was a good predictor of memory, such that words that took longer to be retrieved were remembered better. Importantly, that was the case only when generation was required: In the no-generation (identity) condition, processing time was not related to recognition memory performance. This work has shown that generation affects conceptual/lexical representations, making an important contribution to the understanding of the relationship between memory and language.

Memory and language are tightly linked. For instance, we have memory representations of the contents of conversations, concerning the facts and events mentioned, but also, often, of the words and phrases used. Additionally, language influences memory: Participants in pair studies remember their own utterances better than their interlocutors’ (e.g., Fischer, Schult, & Steffens, 2015; Hoedemaker, Ernst, Meyer, & Belke, 2017; Yoon, Benjamin, & Brown-Schmidt, 2016). Despite this relationship, memory and language are often researched independently. Our goal is to investigate these domains in tandem, exploring the influence of language production on memory. We do so by examining how picture naming, and the time it requires, influences memory for the picture names.

In an earlier study, we asked participants to name pictures with either the picture names or scrambled letter fragments superimposed and later tested their recognition of the pictures (Zormpa et al., 2019). Pictures with the scrambled letters superimposed were remembered better than ones with the picture names superimposed. This can be interpreted as a *generation effect* (Slamecka & Graf, 1978): When participants generated picture labels themselves, they remembered the pictures better than when the correct names were provided. We interpreted this finding under a distinctiveness account (Hunt & Worthen, 2006). Active generation of the names creates an additional episodic memory trace, which aids picture recognition. This framework has successfully explained a range of phenomena in episodic and visual memory, including the picture superiority effect (Paivio, Rogers, & Smythe, 1968) and the production effect (MacLeod, Gopie, Hourihan, Neary, & Ozubko, 2010).

Our previous study showed the influence of language production on memory, but it did not allow us to determine what level of representation benefits from generation. Providing a picture label reduces the need to perform object identification, a time-consuming part of picture naming (Indefrey & Levelt, 2004). Therefore, one might expect visual features to be less distinctive in memory when the picture name is provided than when it is not. Another possibility is that generation affects later conceptual and/or lexical processes. For instance, as more time is spent looking at a picture during object recognition and name retrieval, more conceptual and lexical information may become activated. In other words, episodic conceptual and lexical representations may be encoded more strongly when picture labels are generated than when they are provided, leading to more distinctive memory representations. Effects may also arise at multiple levels and interact. To begin to distinguish between these possibilities, we conducted a cross-modal version of our earlier experiment: During study, we presented pictures to be named, but during test, we presented the picture names to be recognized. We reasoned that, as the pictures were not shown again, any generation effect could not be due to better memory for the visual properties of the stimuli. Format changes between training and testing have been shown to not affect memory phenomena like the picture superiority effect (Borges, Stepnowsky, & Holt, 1977) and the production effect (Mama & Icht, 2016), but the generation effect reported in Zormpa et al. (2019) was only established when both training and test stimuli were pictures.

We used a study–test paradigm involving three study conditions and a yes/no recognition memory task 24 hours later. The study phase was a self-paced picture-naming task where participants heard primes immediately preceding the targets. There were three prime types: the *identity* prime was the target word itself, the *backward* prime was backward speech, and the *unrelated* prime was a word semantically and phonologically unrelated to the target. This paradigm differed from Zormpa et al. (2019) in that testing was delayed and that the primes were auditory (not written). Both changes made the task harder, ensuring performance would not be at ceiling. In addition, primes were now presented before the picture, allowing participants to concentrate on processing the pictures when they appeared. Based on earlier work (e.g., Schriefers, Meyer, & Levelt, 1990), we expected slower naming in the unrelated compared with the backward prime condition because of competition between the targets and unrelated words. This increase in processing time^1^ should lead to improved recognition memory for picture names in the unrelated compared with the backward condition. Although semantically related distractors would have elicited even slower naming, such distractors could influence memory in unexpected ways, confounding the effects of processing time and semantic relatedness.

We expected unrelated distractors to have a small effect, hindering testing of our hypothesis that an increase in processing time at input would benefit recognition memory. Therefore, we recorded the picture-naming latencies during study and predicted that memory would be best for the items that were named the slowest. On study trials, a dot was displayed to the right of the picture. To advance to the next trial, participants fixated the dot and then pressed “Enter.” We measured when participants turned from the picture to the dot (gaze durations), and when they pressed the button (button-press latencies), as indices of exposure time used in exploratory analyses. The self-paced naming task served to ensure that the latency measures reflected the time needed for each trial, and the surprise memory task served to avoid rehearsal of the pictures during naming.

## Method

### Participants

Sixty individuals (18 male, mean age 22.62 years; range: 18–30 years) participated in this experiment. Eight participants were excluded—two due to technical problems during study and six for not completing the test phase. One additional participant was excluded because of substantially lower hit rates (approx. 25%) than the other participants. This left data from 51 individuals. Participants were recruited from the Max Planck Institute participant database and received 8€. All were native Dutch speakers with normal or corrected-to-normal vision; none reported speech or language problems. A power analysis using an effect size of 3% from Zormpa et al. (2019) showed that 48 participants would provide sufficient power to answer our main research question. Ethical approval was given by the Ethics Board of the Social Sciences Faculty of the Radboud University.

### Materials and design

Stimuli were composed of 246 color pictures from the BOSS database (Brodeur, Dionne-Dostie, Montreuil, & Lepage, 2010; Brodeur, Guérard, & Bouras, 2014) presented in 250 × 250 pixel resolution against a light-gray background (RGB: 153, 153, 153). Match software (van Casteren & Davis, 2007) was used to split the pictures into two sets (A and B) matched on name agreement (*M*_A_ = .93, *M*_B_ = .95), familiarity (*M*_A_ = 4.36, *M*_B_ = 4.39), visual complexity (*M*_A_ = 2.36, *M*_B_ = 2.35), manipulability (*M*_A_ = 2.87, *M*_B_ = 2.88), log_10_ word frequency (*M*_A_ = 2.15, *M*_B_ = 2.22), and duration (ms; *M*_A_ = 683, *M*_B_ = 679). Familiarity, visual complexity, and manipulability scores were extracted from the BOSS database, frequency scores from the SUBTLEX-NL database (Keuleers, Brysbaert, & New, 2010), and name agreement scores were collected from six native Dutch speakers that did not participate in the experiment. Either Set A or Set B was presented at study; the condition in which they appeared was counterbalanced across six lists. All 246 images were presented at test, such that for three lists, Set A served as foils and for the other three Set B served as foils.

At study, participants heard a label before each picture^2^ (duration 285–1,234 ms), recorded by a female native Dutch speaker. In the identity condition, primes were the picture names. In the backward condition, primes were pseudorandomly selected foils played backwards, created using the “Reverse” command in Praat (Boersma & Weenink, 2018). None of the backward foils sounded like the targets. In the unrelated condition, primes were semantically and phonologically unrelated Dutch words. Semantic relatedness was judged by a native Dutch speaker. Phonological relatedness was determined by Levenshtein distance. There was no more than 33.3% overlap between targets and unrelated primes on either measure. Unrelated primes were matched between lists, each appearing for a Set A and a Set B item.

### Apparatus and procedure

The study phase was a picture-naming task conducted at the Max Planck Institute for Psycholinguistics in a soundproof booth with comfortably dim constant lighting. The experiment was controlled using Presentation (Neurobehavioral Systems) and displayed on a 24-in. monitor (1,920 × 1,080 pixel resolution). The right eye was tracked using an EyeLink 1000 Desktop Mount eye tracker (SR Research) sampling at 500 Hz. A head stabilizer was used to minimize head movements and maintain constant distance between participants’ eyes and the screen (54 cm from the end of the camera to the distal end of the chin-rest pad). The table height was adjusted for each participant.

The experiment began with random-order nine-point calibration and validation routines. Trials began with a drift check, followed by a white fixation cross displayed on the center-left of the screen (coordinates 480, 540) for 700 ms. Then participants heard an audio prime (Sennheiser headphones). At the offset of the prime, a picture replaced the fixation cross and a dot appeared at the center-right of the screen (coordinates 1440, 540). Both remained on-screen until the trial ended. After participants named the picture, they looked at the dot and pressed “Enter” to move to the next trial. If they had not fixated the dot for minimally 50 ms, nothing happened when pressing “Enter”; this routine served to dissociate gaze duration on the picture from total trial time.

Before the experimental trials, participants completed 12 practice trials (four per condition) that provided an opportunity for questions; no feedback was given after this point. Trials from the three conditions were intermixed and presented in a unique random order for each participant. The session lasted 20–25 minutes.

The test phase was a self-paced yes/no memory task conducted online using the LimeSurvey (Version 3.14.8) platform. Links and unique tokens were e-mailed to participants 20 hours after the study phase. Participants had 8 hours^3^ to complete the task and were sent reminders after 4 and 6 hours if needed. Participants saw 246 words (123 targets, 123 foils), one at a time, and were instructed to press “Ja” (Yes) for words used at study and “Nee” (No) for the remaining words. They moved to the next trial by pressing “Volgende” (Next). The session lasted 10–15 minutes and was followed by debriefing.

### Analysis

The main dependent variable was memory performance. *Yes* and *no* responses (coded as 1 and 0, respectively) were analyzed using mixed-effects logistic regression. This mirrors standard signal detection analysis while accounting for participant and item variability (DeCarlo, 1998). The main predictors were prime condition and naming latency. All analyses with prime condition as a predictor were run on a data set containing targets only, as foils did not belong in a prime condition. Latency measures were centered and log-transformed (natural log) to resolve convergence issues. The output of the models with centered or logged and centered latencies followed the same pattern. Contrasts for predictors are described below for each analysis. Unless otherwise specified, analyses were preregistered on the Open Science Framework (https://osf.io/sqad9/).

Analyses were run using the lme4 package (Version 1.1-18-1; Bates, Mächler, Bolker, & Walker, 2015) in R (Version 3.5.0; R Core Team, 2018) with the optimizer BOBYQA (Powell, 2009). Initially, the maximal models were fit and then reduced to overcome convergence problems or overfitting (correlations exceeding .95). Reported *p* values were obtained from maximum likelihood tests comparing a full model with one without the effect of interest. Reported 95% confidence intervals were calculated using the *profile* method of the *confint* function.

Trials were excluded when participants failed to name a picture, when they named it with an unexpected word, or when they repeated a word (9% of the data). This includes naming two pictures with the same word (e.g. “doughnut” for a doughnut and a bagel) and naming a target (e.g., a shark) using the name of a foil in the memory task (“dolphin”).

## Results

Participants’ memory was generally accurate (*M* = 79%, *SD* = 41%). The first analysis examined the effect of probe type (target vs. foil) on memory performance, as measured by *yes* responses in the memory task, to assess overall accuracy and response bias.^4^ This analysis was run separately from the prime condition analysis, as the design of this study was not fully crossed—that is, foils did not appear in a prime condition. Probe type was sum-to-zero contrast-coded (targets = .5, foils = −.5). The random effects structure included by-participant and by-item intercepts and by-participant and by-item random slopes for probe type.

**Fig. 1:**
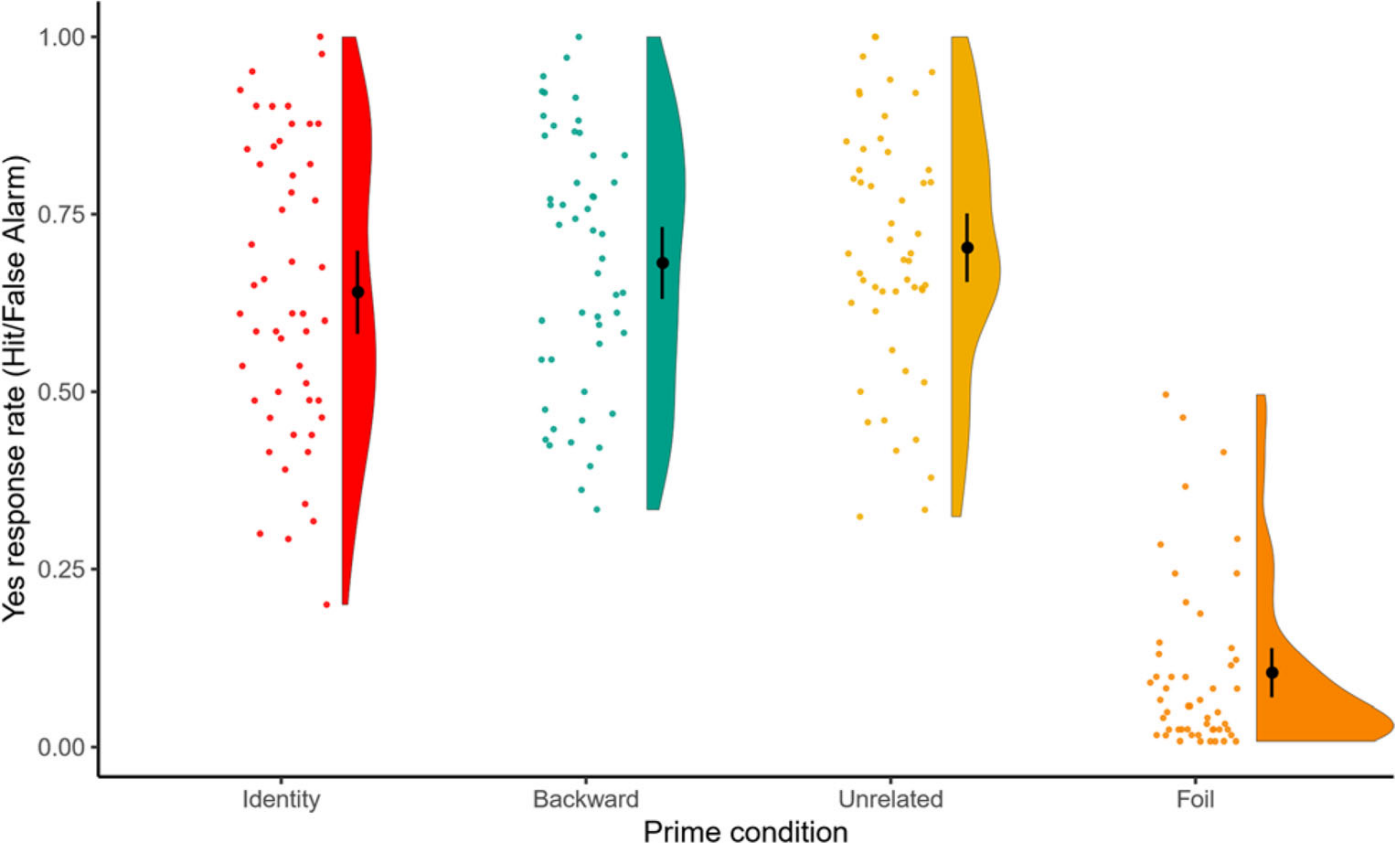
Hit rates by prime condition. The dot represents the condition mean and the bars normalized within-participant 95% confidence intervals. (Color figure online)

Results and a visualization appear in Table 1 and Fig. 1. The significant negative intercept reflects a *no* bias. The significant effect of probe type reflects that participants were more likely to say *yes* to targets than foils—that is, they were highly accurate in differentiating between old and new items.

**Table 1:** Mixed-effects logistic regression testing the effect of probe type (i.e., targets vs. foils) on memory (log-odds of *yes* responses)

*Fixed effects*						*Random effects*			
	Estimate	*SE*	Wald *z*	*p*	CI			Variance	*SD*
Intercept	−.96	.13	−7.24	<.001	−1.23, −.70	Participant	Intercept	.26	.51
Target vs. foil	3.92	.24	16.10	<.001	3.44, 4.42		Target vs. foil	1.28	1.13
						Item	Intercept	.76	.87
							Target vs. foil	2.43	1.56

We then examined the effect of prime condition on memory performance. Prime condition was Helmert coded and split into two contrasts. The first contrast tested the effect of generation by comparing the identity condition (contrast = −0.5) to the average of the backward and unrelated conditions (contrast for both = 0.25), while the second contrast tested the effect of processing time (as a result of competition) by comparing the backward (contrast = −0.5) to the unrelated condition (contrast = 0.5). The random effects structure included by-participant and by-item intercepts and by-participant and by-item slopes for the generation contrast.

Results appear in Table 2 and Fig. 1. The significant positive intercept term reflects a *yes* bias to targets, indicating high accuracy. Hit rates were significantly higher in the backward and unrelated conditions than in the identity condition, showing a memory benefit for generated words. In contrast, hit rates did not differ significantly between the backward and unrelated conditions.

**Table 2:** Mixed-effects logistic regression testing the effects of generation and processing time (as manipulated by prime condition) on memory (log-odds of *yes* responses)

Fixed effects						Random effects			
	Estimate	*SE*	Wald *z*	*p*	CI			Variance	*SD*
Intercept	1.05	.16	6.47	<.001	.73, 1.37	Participant	Intercept	1.10	1.05
Id vs. Bw & Un	.46	.16	2.87	.01	.13, .78		Id vs. Bw & Un	.69	.83
Bw vs. Un	.13	.08	1.53	.13	−.04, .29	Item	Intercept	.75	.87
							Id vs. Bw & Un	.36	.60

*Note.* Id = identity condition; Bw = backward condition; Un = unrelated condition

We then tested how well the three latency measures predicted memory. All measures were positively correlated, with a moderate-to-strong correlation between naming latency and gaze duration (*r* = .46, *p* < .001) and strong correlations between gaze duration and button-press latency (*r* = .59, *p* < .001) and between naming and button-press latency (*r* = .65, *p* < .001).

The prime conditions were associated with different average naming times: Participants were approximately 300 ms faster in the identity condition than in the backward and unrelated conditions due to repetition priming (see Table 3). As such, naming latencies should also be a good predictor of subsequent memory. Additionally, since the three latency measures were highly correlated, the same should hold for the other latency predictors.

**Table 3:** Means (and *SD*s) for naming latency, gaze duration, and button-press latency for each prime condition

	Naming latency	Gaze duration	Button-press latency
Identity	656.43 (172.95)	1,138.44 (593.19)	1,426.84 (526.46)
Backward	965.93 (355.02)	1,423.51 (659.36)	1,757.30 (626.35)
Unrelated	983.90 (402.44)	1,433.74 (694.92)	1,777.36 (658.16)

The differences between the prime conditions led us to run two nonpreregistered analyses. The first tested the combined effects of naming latency and prime condition on memory performance, with both terms entered as fixed effects. Trials on which participants hesitated or stuttered were excluded (.027% of the data). The random effects structure included by-participant and by-item intercepts as well as by-participant and by-item slopes for naming latency.

**Fig. 2:**
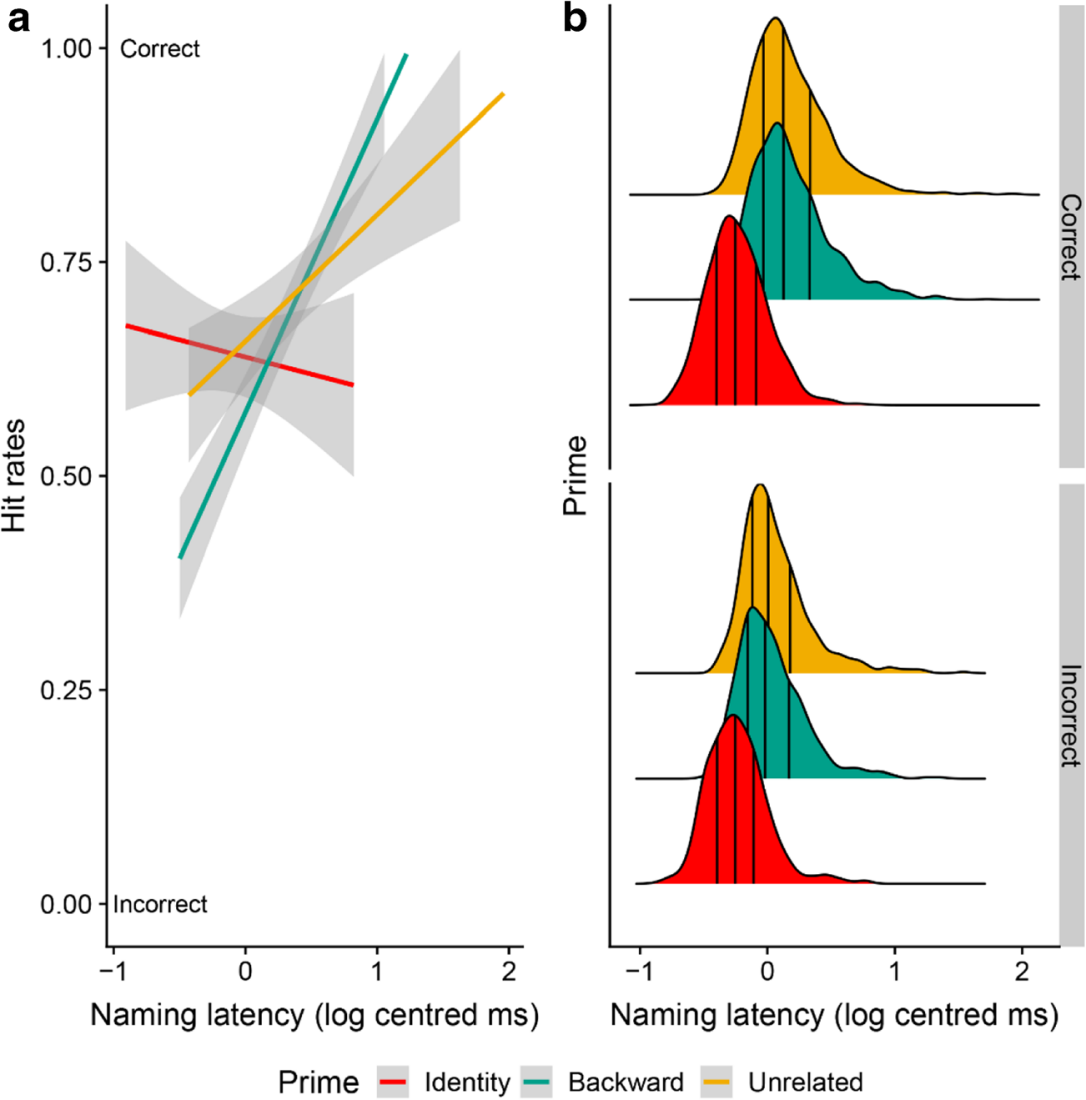
**a** Hit rates by prime condition and naming latency. Naming latency was binned to the second decimal point to calculate hit rates. **b** Stacked density plots for each prime condition of correct (top) and incorrect (bottom) responses by naming latency. The lines signify the first, second, and third quartile. (Color figure online)

Naming latency was a significant predictor of memory performance, with longer naming times associated with better memory (see Table 4). There were no significant main effects of prime condition. However, both contrasts created from prime condition interacted significantly with naming latency. In the identity condition, there was no effect of naming latency on memory performance. As Fig. 2 shows, these trials were remembered relatively poorly regardless of time spent on naming. In contrast, in both the backward and unrelated conditions, longer naming latencies led to improved memory performance. A cross-over interaction between the backward and unrelated conditions was also observed, such that for the slowest trials, the backward condition led to better memory performance than the unrelated condition, while for the fastest trials, the unrelated condition led to better memory performance than the backward condition.

**Table 4:** Mixed-effects logistic regression testing the effect of prime condition and naming latency on memory (log-odds of *yes* responses)

Fixed effects						Random effects			
	Estimate	*SE*	Wald *z*	*p*	CI			Variance	*SD*
Intercept	.89	.16	5.61	<.001	.57, 1.20	Participant	Intercept	1.04	1.02
Naming Latency	1.26	.07	5.74	<.001	.84, 1.72		Nam Lat	.59	.77
Id vs. Bw & Un	.08	.13	.60	.55	−.18, .33	Item	Intercept	.60	.78
Bw vs. Un	.17	.09	1.92	.057	−.01, .35		Nam Lat	.70	.84
Nam Lat:Id vs. Bw & Un	2.30	.41	5.68	<.001	1.51, 3.13				
Nam Lat: Bw vs. Un	−.68	.33	−2.08	.04	−1.34, −.02				

*Note.* Id = identity condition; Bw = backward condition; Un = unrelated condition; Nam Lat = naming latency and has been log-transformed and centered

The second analysis added gaze and button-press measures to the model including naming latency and prime condition to examine whether they predicted any additional variance. The gaze duration data were extracted from the time window between picture onset and the end of the trial. As seen in Table 5, naming latency was a better predictor of memory (*β* = 1.18) than either gaze duration (*β* = −.03) or button-press latency (*β* = .17). Furthermore, compared with the original model, model fit did not significantly improve by adding either gaze duration (*p* = .78) or button-press latency (*p* = .36).

**Table 5:** Mixed-effects logistic regression testing the effect of naming latency, prime condition, gaze duration, and button-press latency on later memory performance (log-odds of *yes* responses)

Fixed effects						Random effects			
	Estimate	*SE*	Wald *z*	*p*	CI			Variance	*SD*
Intercept	.88	.16	5.65	<.001	.58, 1.20	Participant	Intercept	1.03	1.01
Naming latency	1.18	.24	4.81	<.001	.70, 1.68		Nam Lat	.60	.78
Gaze duration	−.03	.09	−0.28	.78	−.20, .15	Item	Intercept	.60	.77
Button-press latency	.17	.18	.92	.36	−.19, .52		Nam Lat	.71	.84
Id vs. Bw & Un	.08	.13	.65	.52	−.17, .34				
Bw vs. Un	.171	.09	1.91	.06	−.01, .35				
Nam Lat: Id vs. Bw & Un	2.29	.41	5.65	<.001	1.50, 3.12				
Nam Lat: Bw vs. Un	−.68	.33	−2.08	.04	−1.35, −.02				

*Note.* All latency measures have been log-transformed and centered. Id = identity condition; Bw = backward condition; Un = unrelated condition. Nam Lat = naming latency

## Discussion

Our results demonstrate that naming pictures, compared with repeating their names, leads to superior recognition memory for the picture names. This is consistent with the generation effect in Zormpa et al. (2019). The finding that generation improves recognition memory even when picture names are used at test indicates a postvisual origin of the effect: If generation enhanced only the visual representation of the pictures, then no generation effect should be found in a test using picture names. A generation effect for picture labels, as observed in this study, can arise only if the representations that generation enhances are conceptual or lexical in nature.

The study phase of the experiment was self-paced; as such, study time variations might account for the observed memory benefit for generated words. As expected, pictures were named faster when they required no generation. If longer processing time leads to more distinctive episodic representations, then this would predict worse recognition for items studied for less time. Indeed, an analysis including naming latency as a predictor along with prime condition showed no main effect of prime condition. Thus, processing time accounts for substantial variance in memory performance.

However, the observed interaction between naming latency and prime condition suggests that additional time benefits memory only when it is spent preparing to name. That is, when generation was not required (identity condition), participants were poor at recognizing picture names regardless of how long it had taken them to repeat them. Increasing time spent preparing to repeat a picture label does not affect memory.

In contrast, when generation was required (backward and unrelated conditions), longer naming time was associated with better memory. In these conditions, variations in naming time likely reflect variations in conceptual and lexical processing, showing an important link between psycholinguistic processes and memory. This claim is further supported by the exploratory analysis adding gaze duration and button-press latency to a model including naming latency: Neither explained any additional variance, indicating that it is specifically time spent preparing to name a picture that improves memory performance, not simple exposure time.

To conclude, we have replicated the generation effect in a cross-modal format, demonstrating that the generation effect observed in picture naming derives from enhanced conceptual and linguistic representations. This is important for theories of episodic memory in language, as language involves reference to objects from past contexts. Our results underscore why this might be easier for speakers than listeners: Generating a name leads to a better episodic representation of the associated concept and linguistic features. This informs research on the intersection of memory and language with implications for phenomena like pronominal resolution and common ground building.

### Author note

We thank Maarten van den Heuvel for his assistance with programming the experiment and Annelies van Wijngaarden and the student assistants of the Psychology of Language department for their help with translations and stimuli creation.

### Open practices statement

This experiment was preregistered (https://osf.io/sqad9/); materials and data are available at https://osf.io/eqzcu/.

## Footnotes

1 In our preregistration, we conceptualized this as cognitive effort and collected pupil sizes for an exploratory study reported at: https://osf.io/w39gu/. However, we were only able to measure processing time, so we restrict our discussion to that.

2 In an earlier experiment, with written primes presented 900-1500 ms before the picture, the generation effect disappeared (https://osf.io/5xe8f/). This may be due to the match between study and test in the identity condition: In this condition, participants saw a written word which they later had to recognize, whereas in the other two conditions participants produced a word in the spoken modality and later had to recognize it in the written modality. Alternatively, the long delay between prime and target may have separated them in two episodes, thus enhancing memory.

3 Due to technical problems, two participants performed the task 9 hours and 10 hours later.

4 The preregistered analysis used accuracy, not *yes* responses. That model had convergence problems when calculating 95% CIs, presumably due to invariance in the dependent variable. This was solved by using *yes* responses.
